# An Unexpected Case Report of Adrenal Lymphangioma: Mimicking Metastatic Tumor on Imaging in a Patient With Pancreatic Cancer

**DOI:** 10.3389/fendo.2020.610744

**Published:** 2021-01-06

**Authors:** Shang Wan, Xijiao Liu, Bole Tian, Dan Cao, Mao Li, Yuhao He, Bin Song

**Affiliations:** ^1^ Department of Radiology, West China Hospital of Sichuan University, Chengdu, China; ^2^ Department of Pancreatic Surgery, West China Hospital of Sichuan University, Chengdu, China; ^3^ Department of Oncology, West China Hospital of Sichuan University, Chengdu, China; ^4^ Department of Neurosurgery, Chengdu Third People’s Hospital, Chengdu, China

**Keywords:** adrenal tumor, lymphangioma, pancreatic cancer, magnetic resonance imaging, computed tomography, differential diagnosis

## Abstract

Adrenal lymphangioma is a very rare benign lesion worldwide and remains challenging for early diagnosis, especially when the patient has some complicated clinical disease. This is an unusual case of a 68-year-old man who was admitted to our hospital with a history of pancreatic tumor. Computed tomography (CT) images and subsequent magnetic resonance imaging (MRI) revealed a mass located in the left adrenal gland, presenting a similar enhancement pattern of the pancreatic tumor, and according to the imaging features, the patient was suspected to have an adrenal metastatic tumor originating from the pancreatic tumor. The patient underwent a surgical resection of the pancreatic tumor and the left adrenal gland. The pathologic diagnosis proved to be lymphangioma deriving from the left adrenal gland. This is the first report presenting an atypical adrenal lymphangioma mimicking a metastatic tumor of pancreatic origin, which might be suggestive in the diagnosis of adrenal lesions and the subsequent clinical treatment, especially when patient has a particular medical history. As we know, imaging examination is helpful for accurate preoperative diagnosis; however, the diagnosis of malignant tumor solely based on imaging procedures should be made cautiously by radiologists.

## Introduction

Adrenal lymphangioma is a very rare benign lesion generally deriving from the lymphatic endothelial cells (1). Most lymphangiomas are located in the neck and axilla (1) and are found occasionally on images without specific clinical symptoms (2). The pathologic diagnosis of the postoperative is regarded as the definitive diagnosis. Therefore, it remains a diagnostic difficulty to distinguish these lesions from some malignant tumors before surgery or biopsy. We herein present a rare case of left adrenal lymphangioma that was primarily misdiagnosed to be a metastatic tumor of pancreatic origin owing to imaging findings and clinical history. Finally, it was pathologically confirmed to be a lymphangioma originating from the adrenal gland.

## Case Report

A 68-year-old gentleman was referred to our hospital with a medical history of pancreatic tumor associated with a left adrenal mass for more than 4 months. Before hospital admission, this patient underwent an MRI scan and an ultrasound-guided biopsy in another hospital. The MRI finding indicated a pancreatic tumor and a left adrenal mass, which was considered to be of a pancreatic origin. The pancreatic biopsy revealed a small number of adenocarcinoma cells. The patient was referred to our hospital for further treatment. Before this hospitalization, he had two cycles of chemotherapy in our hospital to treat the pancreatic tumor and the adrenal mass. At the same time, several CT scans were performed, and the CT findings also supported the left adrenal mass as a metastatic tumor.

**Figure 1:**
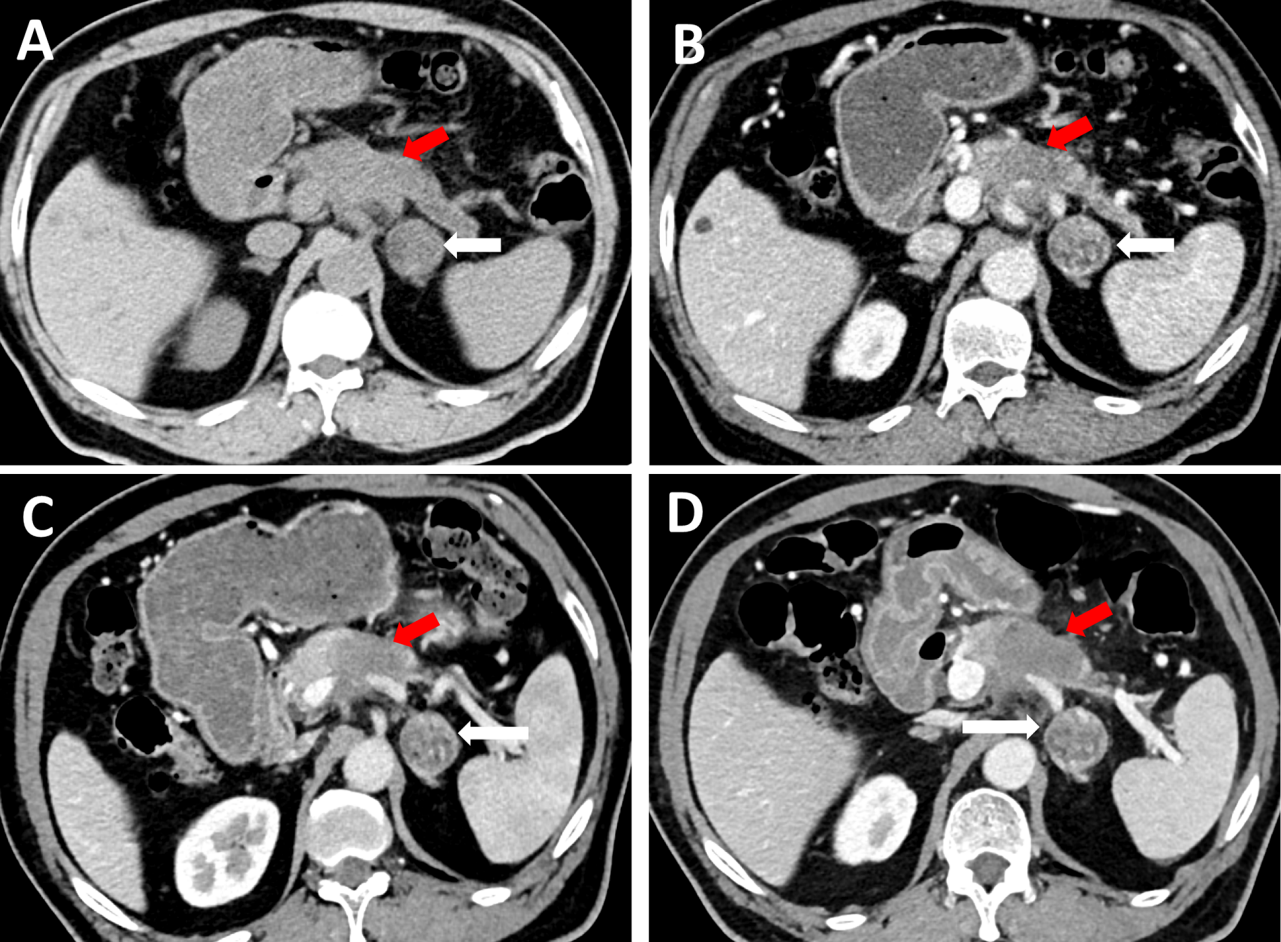
On CT, a slightly low-density and ill-defined mass in the body of pancreas was detected on plain CT scan (**A**, red arrow), with a size of 4.1 cm × 2.8 cm, likewise, a left adrenal lesion measuring 2.8 cm × 2.8 cm can be observed, with relatively well-defined margin and mixed-density (**A**, white arrow). The left adrenal lesion showed continuously nonuniform enhancement in the portal venous phase (**B**, white arrow), the same enhancement pattern can be observed in the pancreatic tumor (**B**, red arrow). When compared with the previous CT scans 1 month ago (**C**, portal venous phase) and 3 months ago (**D**, portal venous phase) respectively, the size and density of pancreatic tumor (red arrow) and adrenal lesion (white arrow) presented no significant changes through the whole timeline.

**Figure 2:**
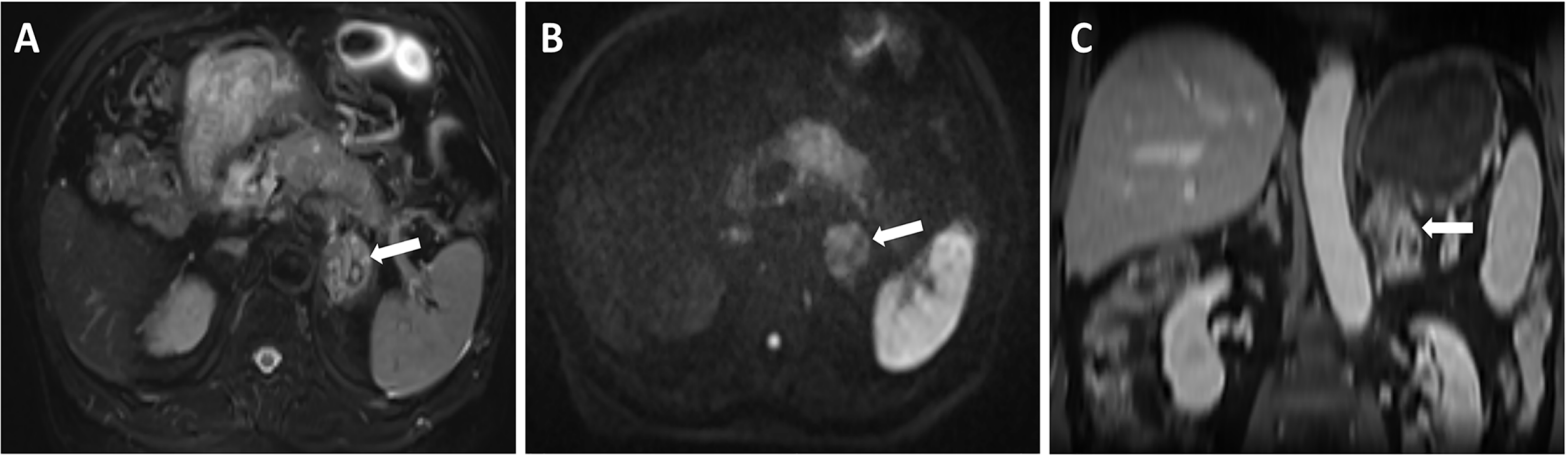
On MRI, T2-weighted sequences showed a mixed hyperintense lesion located in the left adrenal **(A)**, and partly restricted diffusion in DWI **(B)**, in contrast-enhanced T1 weighted sequences, this lesion manifested a thin capsular-rim arterial phase hyperenhancement with slow heterogeneous centripetal enhancement **(C)**.

Physical examination after admission revealed an abdominal pain that was aggravated after eating and radiating to the back. He had not experienced any headaches, palpitations, nausea, and emesis, and the blood pressure is within the normal range. The initial workup showed a slightly elevated carbohydrate serum antigen 19-9 (CA19-9) level of 59.8 U/ml (<22 U/ml) and an elevated carbohydrate serum antigen 125 (CA125) level of 90.2 U/ml (<35 U/ml). Additional blood laboratory tests revealed an increased platelet count level of 538 × 10^^9^/L (100–300 × 10^^9^/L) and a slightly elevated white blood cell count level of 10.14 × 10^^9^/L (3.5–9.5 × 10^^9^/L). Liver function test revealed a slightly decreased total bilirubin level of 4.6 umol/L (5.0–28.0 umol/L) and a decreased albumin level of 37.9 g/L (40–55 g/L), along with a decreased uric acid level of 152 umol/L (240–490 umol/L). Plasma pancreatic enzyme revealed a significantly elevated lipase level of 4,699 IU/L (13–60 IU/L) and an elevated amylase level of 341 IU/L (35–135 IU/L); plasma epinephrine and norepinephrine were both within the normal range, and plasma aldosterone and renin activity were not tested. To evaluate the pancreatic tumor and the left adrenal mass, an abdominal CT scan was performed, which showed a slightly low-density (18–38 HU, plain scan) and ill-defined mass in the body of the pancreas (**Figure 1A**), with a size of 4.1 × 2.8 cm. Simultaneously, it revealed a left adrenal lesion with relatively well-defined margin and mixed density (−18 to 46 HU, plain scan) (**Figure 1A**), with a size of 2.8 × 2.8 cm. Both the pancreatic and the adrenal masses showed a continuously nonuniform enhancement in the portal venous phase (**Figure 1B**). The size and density of pancreatic and adrenal lesions showed no significant changes during different phases of chemotherapy compared with the previous CT scans in our hospital (**Figures 1C, D**). The patient then underwent MRI to more precisely evaluate these lesions; on T2-weighted sequences, it showed a mixed hyperintense lesion in the left adrenal gland with a size of 2.8 × 2.8 cm (**Figure 2A**) and partly restricted diffusion in diffusion-weighted imaging (DWI) (**Figure 2B**). After intravenous administration of extracellular contrast agent, this lesion manifested a thin capsular-rim arterial phase hyperenhancement with slow heterogeneous centripetal enhancement (**Figure 2C**).

Both CT and MRI features supported the malignant suspicion of the adrenal lesion. The surgical resection of the left adrenal gland followed by histological examination was elected for the final diagnosis. Hence, the upper midline laparotomy was performed, the body and tail of pancreas together with the left adrenal gland and spleen were removed, and the tumor of the adrenal was measured at 4 × 3.5 cm during surgery. The postoperative course was uneventful and the patient was discharged on the 25th postoperative day. The patient was followed up by CT scan 3 months after discharge and was asymptomatic, no recurrence sign of pancreatic and adrenal lesions was found.

**Figure 3:**
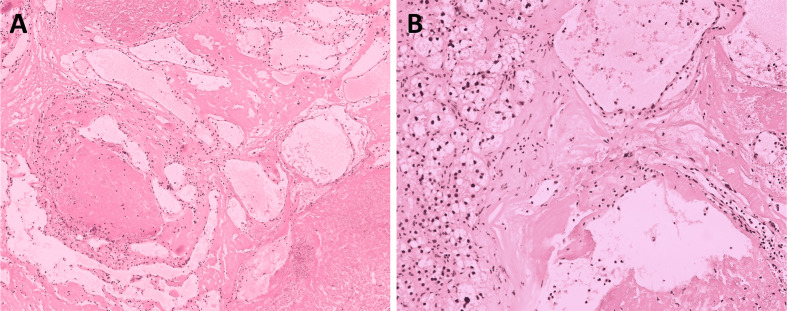
Histological view at low magnification **(A)** and high magnification **(B)** showing a multi-cystic lesion of the left adrenal (hematoxylin and eosin staining, ×100 and ×200), the entire picture is consistent with benign lymphangioma.

The pathologic diagnosis of pancreatic specimens confirmed pancreatic ductal adenocarcinoma. The specimen from the adrenal gland showed a solid mass with an irregular appearance, measuring 3 × 2 × 0.4 cm, and the definitive diagnosis confirmed a lymphangioma originating from the left adrenal gland (**Figure 3**), which indicated its benign nature and excluded the possibility of a metastatic tumor. The lymphangioma tumor was positive for CD31, CD 34, and D2-40 and negative for pan-cytokeratin (PCK) immunohistochemically, and the definitive diagnosis of a left adrenal lymphangioma was confirmed.

## Discussion

This is a rare case of lymphangioma originating from the left adrenal gland, with a specific medical background of pancreatic cancer. Till now, this is the first case report presenting such lesion accompanying this type of medical history, and this adrenal lesion was primarily considered as a metastatic pancreatic tumor, with the malignant indicative imaging features on CT and MRI. Fortunately, the patient underwent a successful pancreatectomy and adrenalectomy, and the follow-up CT scan revealed no signs of recurrence.

Although these entities of lymphangiomas are commonly thought of as benign lesions mostly located in the neck, axilla, and mediastinum (1, 3), the exact pathogenesis still remains unclear. The abnormal development of the lymphatic tissue, which leads to lymphatic vessel ectasia, is the most widely accepted etiology (1, 4, 5). Previous cases reported that this type of lesions can occur in both adrenal glands and the right side is more often affected (1, 6). Our case presented a lesion located in the left adrenal gland. Notably, contrary to this case of an elderly male patient in our report, most adrenal lymphangiomas occur in children and women (7), with a mean age of 39.5 (16–60) years (5).

Most adrenal lymphangiomas are asymptomatic lesions without specific medical backgrounds. They are usually found incidentally and patients may present with some nonspecific clinical manifestations such as abdominal and back pain, because of the tumor size or the position of the lesion (1, 7). In this case presentation, we found that this patient had a medical history of pancreatic tumor on radiological images, along with abnormal laboratory test results (elevated CA19-9 and CA125) that supported the risk of pancreatic cancer, and the postoperative pathologic finding confirmed the diagnostic suspicion, which may be one of the diagnostic dilemmas for the benign lesion of adrenal lymphangioma.

Imaging modalities are valuable diagnostic assets for lymphangiomas and may sometimes help characterize these lesions (8, 9). Although these imaging features lack specificity, the ultimate diagnosis relies on the definitive pathology (9). Of note, with its multiparametric and multifunctional imaging features, MRI is considered to be more specific than CT scan to distinguish some malignant masses from benign ones (8) and may contribute to the preliminary qualitative diagnosis. Moreover, some complications, such as intralesional hemorrhage, can be easily detected on MRI. Generally and in most reported cases, adrenal lymphangiomas are asymptomatic nonfunctioning lesions and show cystic imaging features. On CT images, they are usually hypodense with internal, well-defined, and capsular enhancement (8, 10), and on MRI, these lesions are commonly hypointense and non-enhancing on T1-weighted images and homogeneously hyperintense on T2-weighted images (8, 10). However, in this case, ill-defined, mixed intensity and density, non-uniform enhancement, and partly restricted diffusion in DWI were observed on CT and MRI, which were generally deemed a substantial risk of malignancy and were not consistent with typical imaging manifestations of the cystic nature of lymphangioma. These atypical imaging features and the specific medical history may lead to misdiagnosis and make diagnostic process even more difficult.

Differential diagnosis of the adrenal lymphangiomas reported in this literature mainly refers to metastatic tumors, primary adrenal tumors, angiosarcomas, multi-cystic mesotheliomas, or adrenal cysts (4). Phaeochromocytomas or paragangliomas of the primary adrenal tumors, particularly, should be noted in the differential diagnosis of the disease, nowadays, the most accurate way to biochemically detect or exclude a phaeochromocytomas or paragangliomas is with a significant elevated plasma or 24 h urine metanephrines/normetanephrines, rather than the blood norepinephrine and epinephrine (11, 12). However, in this case report, only blood norepinephrine and epinephrine were tested (both were within the normal range), which are inadequate for the evaluation of an adrenal lesion (such as pheochromocytoma, lymphangioma), because the imaging findings lack specificity, the hormone testing for biochemical markers would be of great value and a comprehensive biochemical evaluation for this patient are warranted.

Few studies reported adrenal lymphangiomas with clinical manifestation, and most lesions were found incidentally during a physical examination (13). In our case, this patient complained a continuous abdominal pain, we are still uncertain whether this type of pain is definitely associated with the adrenal lymphangioma. Although diagnosed by experienced abdominal radiologists, this benign lesion was considered as a metastatic tumor from the pancreas, and it is difficult to distinguish lymphangioma from metastatic tumor only with imaging modalities, especially considering the malignant indicative imaging features of this case.

Histopathology, along with immunohistochemistry, makes the final diagnosis and is regarded as the reference standard (14). In the previous reported literature, lymphangiomas are commonly positive with some specific markers, including CD31 and CD34 (1, 4). Other researches recommended D2-40 to be a more specific marker in the diagnosis of lymphangioma (1, 15). All these markers can be observed in this case presentation.

In the previous series cases of adrenal lymphangiomas, no relevant case with a specific history of pancreatic cancer has been reported. In this case, it was challenging to correctly diagnose a benign lesion of adrenal and may mislead the radiologists given the medical history of pancreatic tumor, along with these atypical imaging findings. In other words, although there are no specific radiological signs yet, we should be aware that adrenal lymphangiomas tend to be cystic-like lesions on images generally (8, 10, 16), which may help with the preoperative diagnosis and guide the clinical treatment. When it comes to an atypical case of such a lesion, for example, the patient in this case, a notable and uncommon differential diagnosis on imaging modalities should be recommended. For the clinicians, an early comprehensive preoperative diagnosis may affect the treatment option; laparoscopic adrenalectomy is usually considered for small to medium size benign ones (17). However, an open adrenalectomy should be suggested for the larger and suspected malignant lesions (14). In this presented case, the patient underwent an open adrenalectomy during the pancreatectomy owing to the suspected malignancy.

In conclusion, this is the first case report presenting an adrenal lymphangioma with a medical background of pancreatic cancer. On the one hand, we hope this type of lesions can be reminded and suggestive in the clinical differential diagnosis; on the other hand, because pathognomonic presentations of adrenal lymphangioma in imaging tools are still lacking, a preoperative diagnosis of malignant tumor should be made cautiously based on imaging modalities, and more relevant evidence and case reports are needed to help us further investigate this disease.

## Data Availability Statement

The original contributions presented in the study are included in the article/supplementary material; further inquiries can be directed to the corresponding author.

## Ethics Statement

The studies involving human participants were reviewed and approved by the West China Hospital of Sichuan University. The patients/participants provided their written informed consent to participate in this study. Written informed consent was obtained from the patient for publication of this case report and any accompanying images.

## Author Contributions

DC, SW, and XL acquired the data. BT, DC, and ML analyzed and interpreted the data. SW and XL conductued the radiological analysis of MRI and CT images, and prepared the manuscript. YH and BS revised the manuscript. All authors contributed to the article and approved the submitted version.

## Conflict of Interest

The authors declare that the research was conducted in the absence of any commercial or financial relationships that could be construed as a potential conflict of interest.

## Abbreviations

CT, computed tomography; MRI, magnetic resonance imaging; HU, hounsefield units; CA19-9, carbohydrate serum antigen19-9; CA125, carbohydrate serum antigen125; PCK, pan-cytokeratin; DWI, diffusion-weighted imaging.
